# Evaluation of Fully Teleoperated Robotic Endovascular Interventions with Haptic Feedback: The SENTANTE Endovascular Robotic System

**DOI:** 10.1007/s00270-026-04375-w

**Published:** 2026-02-25

**Authors:** Emiel W. M. Huistra, Giovanni Federico Torsello, Konstantinos Stavroulakis, Lorenzo Patrone, Arturas Mackevicius, Max Amor, Vilius Dambrauskas, Vaidas Labunskas, Leona Damalakiene, Clark J. Zeebregts, Tomas Baltrunas

**Affiliations:** 1 Division of Vascular Surgery, Department of Surgery, University Medical Center Groningen, University of Groningen, Groningen, The Netherlands; 2 Department of Clinical and Interventional Radiology, Göttingen University Medical Center, Göttingen, Germany; 3 Department of Vascular Surgery, Ludwig Maximilians University, Munich, Germany; 4 Vascular and Endovascular Surgery, Mathias Spital Rheine, Rheine, Germany; 5 Vascular and Endovascular Surgery Unit, San Giovanni Di Dio Hospital, Florence, Italy; 6 Department of Vascular Surgery, Republican Vilnius University Hospital, Vilnius, Lithuania; 7 Faculty of Medicine, Vilnius University, Vilnius, Lithuania; 8 Department of Interventional Cardiology, U.C.C.I. Polyclinique d’Essey, Nancy, France; 9 Health Telematics Science Institute, Kaunas University of Technology, Kaunas, Lithuania

**Keywords:** Robotic surgical procedures, Robotics, Teleoperation, Endovascular, Interventional radiology

## Abstract

**Purpose:**

To evaluate a fully robotic, teleoperated system for performing various endovascular interventions in a preclinical porcine model.

**Materials and Methods:**

The SENTANTE™ Endovascular Robotic System was assessed in six healthy porcine subjects using standard endovascular devices. Outcomes were assessed via procedural monitoring and gross pathological analysis. Haptic feedback performance was qualitatively assessed by the operators using a four-point scale (1 = very good, 2 = fair, 3 = poor, 4 = inadequate).

**Results:**

A total of 18 endovascular interventional procedures were successfully completed, including renal artery stenting (3), embolization of the superior right renal artery branch (3), percutaneous transluminal angioplasty (PTA) of the superior mesenteric artery (3), left vertebral artery stenting (3), contralateral iliac artery PTA (3), and ipsilateral iliac artery stenting (3). All procedures (100%) were completed without manual conversion, including pre and post-procedural angiography, resulting in near-zero operator radiation exposure. The system was compatible with standard guidewires (0.014″, 0.018″, and 0.035″), catheters (2.7–8 Fr), and pushable coils (0.018″ and 0.035″). Post-procedural angiography demonstrated no signs of vessel injury. Minimal vessel trauma was observed in 3 of 16 target vessels during gross necropsy. Haptic feedback was rated as very good in three test models and as fair in the remaining three.

**Conclusions:**

In a preclinical model, the SENTANTE™ system demonstrated the feasibility of performing endovascular interventions via teleoperation, including angiography, stenting, coil embolization, and PTA. Its compatibility with standard devices, haptic feedback, and elimination of operator radiation exposure support progression to clinical feasibility trials.

**Graphical Abstract:**

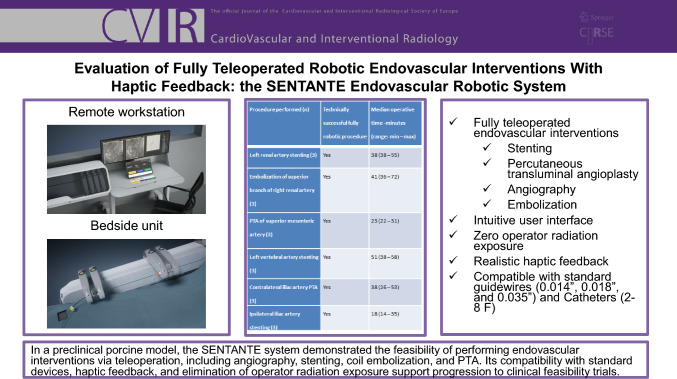

**Supplementary Information:**

The online version contains supplementary material available at 10.1007/s00270-026-04375-w.

## Introduction

Over the past three decades, coinciding with the rise of minimally invasive surgery, robotic telemanipulated systems have gained traction by enhancing surgeon dexterity and precision in laparoscopic procedures [1]. In contrast, progress in endovascular robotics has been more limited [2]. Robotic systems that achieved CE certification for peripheral endovascular interventions included the Magellan™ (Hansen Medical, Mountain View, CA, USA) and the CorPath® GRX System (Siemens Healthineers, Pennsylvania, USA). Both the Magellan and Corpath GRX systems demonstrated the potential benefits of robotic systems in reducing operator radiation exposure, enhanced precision, and enabling teleoperation. However, widespread adoption was limited by challenges related to integration into routine endovascular practice, such as the need for manual interventional device placement, lack of haptic feedback, limited compatibility with standard endovascular devices, and complex user interfaces [3–7].

A novel endovascular robotic system is the SENTANTE^tm^ Endovascular Robotic System (UAB Inovatyvi Medicina, Kaunas, Lithuania). The system consists of a bedside robotic unit capable of manipulating up to three endovascular devices simultaneously: one guidewire and two over-the-wire catheters. The bedside unit is controlled by the operator via teleoperation from a remote workstation. The operator interface consists of standard endovascular devices, mimicking those used at the bedside unit (one guidewire and two over-the-wire catheters). Manipulation of these devices at the remote workstation is replicated in real time by the bedside unit, including angiography and placement of interventional devices. The catheters and guidewire at the workstation provide haptic feedback based on the force measurements obtained at the bedside unit. The aim of the current study was to evaluate the feasibility of performing robotic endovascular interventions using the SENTANTE system.

## Methods

### Study Design

This preclinical study evaluated the performance of a fully teleoperated robotic system for endovascular procedures in a healthy porcine model. All procedures were conducted in a good laboratory practice (GLP)-compliant facility specifically designed for preclinical endovascular research. The robotic system was used to perform a range of diagnostic and therapeutic procedures, including angiography, target vessel stenting, embolization, and percutaneous transluminal angioplasty (PTA). All procedures utilized commonly available catheter-based interventional devices (e.g., guidewires, sheaths, balloons, stents, and coils), which were remotely manipulated, inflated, deployed, and retrieved using the robotic system.

### Population

The porcine model was selected because of the anatomical and dimensional similarity between its cardiovascular system and that of humans [8, 9]. The study animals were randomly assigned to two groups: Group A underwent procedures via right carotid artery access, and Group B via left femoral artery access. Procedures performed through carotid access included angiography, left renal artery stenting, embolization of the superior branch of the right renal artery, and PTA of the superior mesenteric artery (SMA). Procedures performed through femoral access included angiography, left vertebral artery stenting, contralateral iliac artery PTA, and ipsilateral iliac artery stenting. Two access sites, carotid and femoral, were selected to evaluate the system’s ability to navigate different anatomical regions.

### Ethical Considerations and Animal Welfare Compliance

The study was conducted in compliance with ISO 10993–2, GLP standards, and under the Quality Management System of the testing facility. Ethical approval was obtained from the Animal Care and Use Committee of the Testing Facility, which was registered with the CNREEA under the Ethics Committee No. 37. The study was part of the research project ROBOTIQUE 42515. The Program of the Testing Facility conforms with AAALAC International standards as set by the European Convention for the Protection of Vertebrate Animals Used for Experimental and Other Scientific Purposes (Council of Europe ETS 123), French implementing legislation, and the Guide for the Care and Use of Laboratory Animals (NRC 2011).

### Robotic System and Setup

**Fig. 1:**
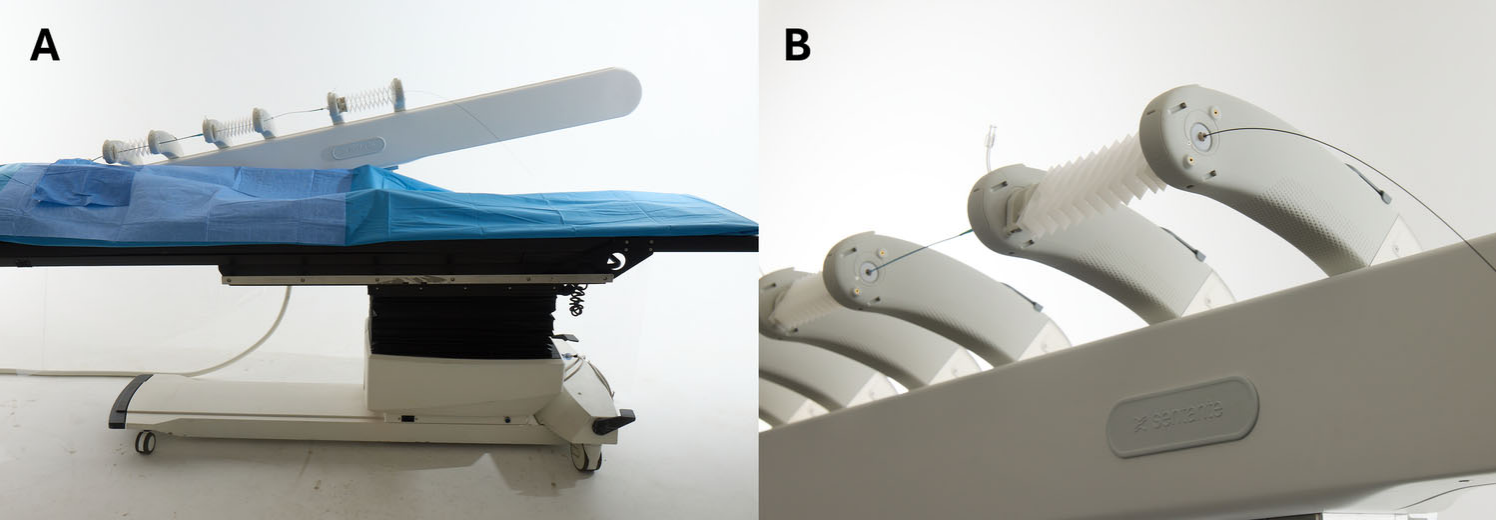
(A/B) The SENTANTE bedside unit inside the operating suite, consisting of three pairs or cassettes that are capable of manipulating three endovascular devices simultaneously (one guidewire and two over the wire catheters) based on operator input from a remote workstation

The robotic system evaluated in this study was the SENTANTE Endovascular Robotic System. The SENTANTE system is designed for the remote delivery and manipulation of standard, commercially available catheter-based devices used in arterial endovascular procedures. The system consists of two main functioning units: a bedside unit positioned adjacent to the patient and a remote workstation outside the radiation field, from which the operator controls the system. The bedside unit is a remotely controlled robotic platform that manipulates the endovascular devices in the patient. In simplified terms, it functions as a rail-based system comprising three pairs of sterile, single-use cassettes, each capable of gripping an endovascular device and moving along the rails to advance or retract the device (Fig. 1A/B). The system can also rotate the devices. The bedside unit enables simultaneous manipulation of up to three coaxial endovascular devices (e.g., one guidewire and two over-the-wire catheters). Standard catheters (2–8 Fr) and guidewires (0.014″, 0.018″, and 0.035″) can be mounted on the bedside unit.

**Fig. 2:**
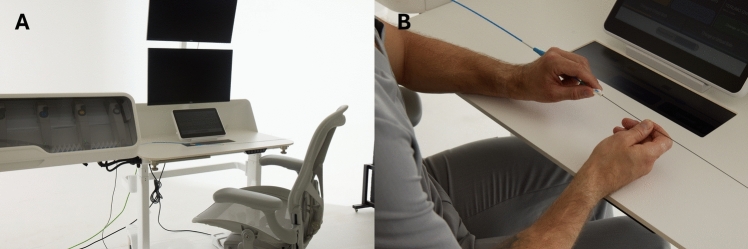
(**A**) The SENTANTE workstation, which delivers the input for the robotic system at bedside from a remote location based on the operator’s movement with the input devices, (**B**) consisting of one guidewire and two over the wire catheters

Endovascular device movement by the bedside unit is determined by the operator input at the workstation. The workstation is equipped with one guidewire and two over-the-wire catheters as the user interface (Fig. 2A and B). The movements made by the operator (advancement, retraction, and rotation of any of the three devices) are replicated by the bedside unit in real-time on a one-to-one basis. Supplemental videos A and B demonstrate both the bedside unit manipulating the endovascular devices and the corresponding operator input at the workstation, respectively. In the current study, the remote workstation was positioned in a separate room adjacent to the operation suite.

Because the robotic system uses standard endovascular devices as its input interface, realistic haptic force feedback is provided to the operator. The bedside unit senses the forces required to manipulate the devices within the patient and relays these forces to the corresponding endovascular devices at the workstation, where the operator feels the same resistance. Conversely, device movement within the patient occurs only when the operator applies an equivalent force to the workstation, thereby matching the force required by the bedside unit to advance or retract the device.

**Fig. 3:**
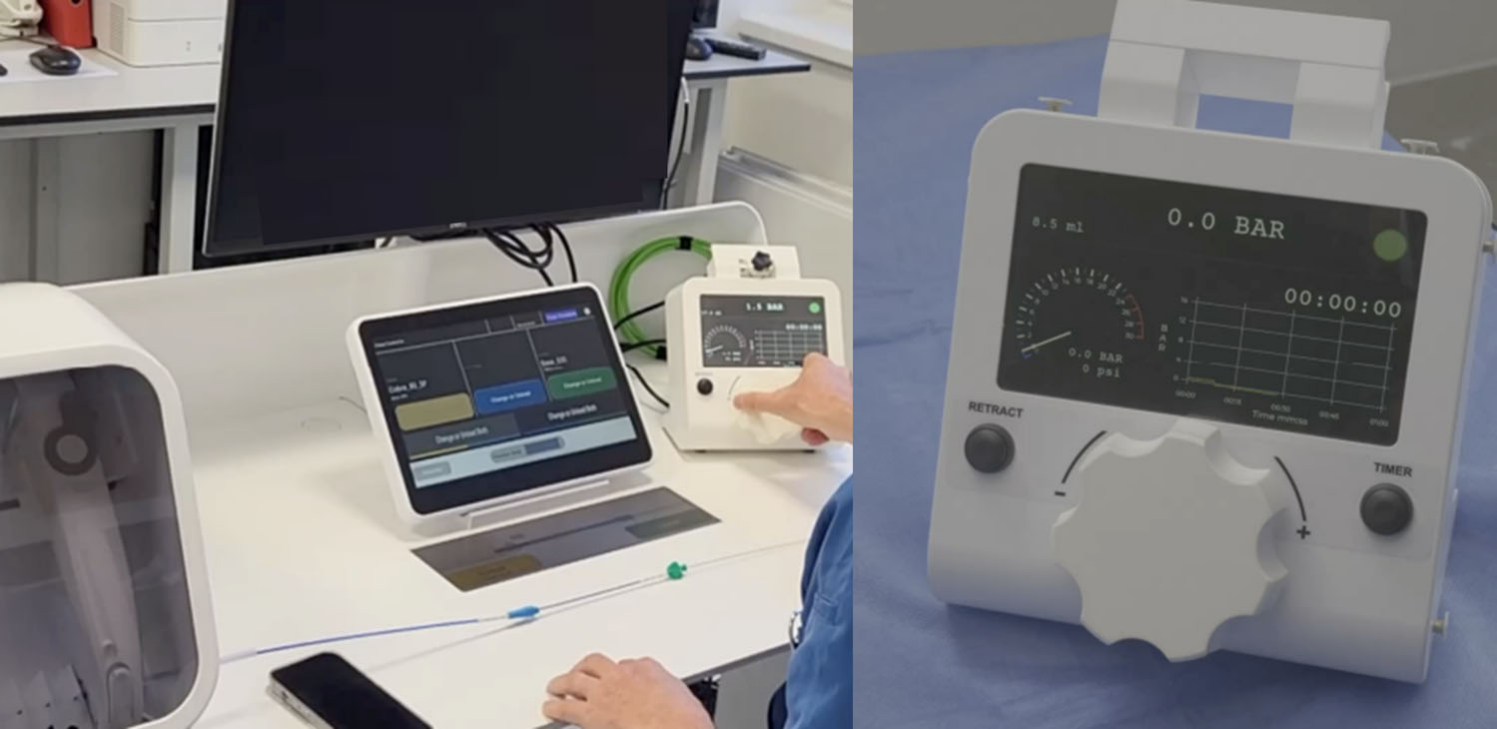
The remote workstation of the SENTANTE is equipped with a system (INFLATE) that allows for remote initiation of balloon inflation or contrast injection by the bedside unit

The workstation is equipped with a system that enables remote balloon inflation and contrast injection by turning a wheel (INFLANTE^tm^; UAB Inovatyvi Medicina, Kaunas, Lithuania) (Fig. 3). This feature allows the operator to perform stenting with balloon expandable stents, PTA, and angiography fully remotely via teleoperation. Catheters and guidewires need to be loaded on the bedside unit manually before advancement or retrieval via the remote workstation. Exchange of endovascular devices that are mounted on the bedside unit (e.g., replacement of the middle catheter with an over-the-wire balloon) is also done manually at the bedside by an assistant.

All instruments and accessories that came into contact with intravascular devices or animal subjects were single-use and sterile. For each procedure, the bedside unit was fully draped in a sterile manner. The robotic system worked independently and in parallel with real-time fluoroscopy and angiographic imaging systems, allowing the operator to visualize the vascular anatomy and device progression throughout the procedure.

### End-points

The primary outcome was technical success. For PTA and stenting procedures, technical success was defined as accurate delivery and deployment of the interventional device at the predefined target location, with expansion to nominal pressure and without signs of vessel injury on post-procedural angiography, including perforation, thrombosis, flow-limiting residual dissection, or distal embolization. For coil embolization procedures, technical success was defined as successful implantation of pushable coils within the intended vessel, resulting in complete occlusion of the target vessel without compromise of adjacent vascular structures or angiographic evidence of vessel injury, including perforation, thrombosis, or flow-limiting residual dissection.

Acute safety of the robotic system was assessed through evaluation of vessel injury, damage to surrounding tissues, general animal health, and complications potentially attributable to device use. In addition, angiographic images were independently evaluated by an external Core Laboratory.

Haptic feedback performance was qualitatively assessed by the operators after completing three procedures on a single model, resulting in a total of six evaluations. Ratings were assigned using a four-point scale (1 = very good, 2 = fair, 3 = poor, 4 = inadequate).

After completion of all interventions, animals were euthanized and a post-mortem evaluation was performed. Target vessels were harvested in full length, from introducer insertion point to the target site, and assessed macroscopically for evidence of vessel trauma or procedural complications. Distal and systemic non-target organs were also harvested and assessed macroscopically for potential off-target effects or device-related injury.

### Statistical Analysis

This study was designed as an exploratory preclinical evaluation and did not include a control group using conventional manual techniques. The aim of the analysis was to descriptively report procedural feasibility, technical success, and acute safety outcomes for each intervention performed with the robotic system. Continuous variables were reported as medians (range: min–max).

## Results

### Study Population

Six healthy porcine subjects (Large White x Landrace crossbreed), approximately four months of age and weighing between 53.5 and 69.5 kg, were included in the study. The animals were evenly assigned to two groups: Group A (*n* = 3) underwent procedures via right carotid artery access, and Group B (*n* = 3) via left femoral artery access. Interventions were performed by five experienced endovascular specialists, including two interventional radiologists, two vascular surgeons, and one interventional cardiologist. Each physician was trained for 30 min on the use of the robotic system prior to performing the procedures.

A total of 18 fully robotic endovascular procedures were successfully completed (Table 1). In Group A, procedures included left renal artery stenting (*n* = 3), embolization of the superior branch of the right renal artery (*n* = 3), and PTA of the SMA (*n* = 3). In Group B, procedures included left vertebral artery stenting (*n* = 3), contralateral iliac artery PTA (*n* = 3), and ipsilateral iliac artery stenting (*n* = 3). For each intervention, pre- and post-angiography was performed robotically without manual assistance.

**Table 1:** Details regarding robotic endovascular procedures in porcine models

Procedure performed	Number of procedures performed	Access site	Technically successful fully robotic procedure*	Median operative time -minutes (range: min – max)
Left renal artery stenting	3	Right carotid artery	Yes	38 (38–55)
Embolization of superior branch of right renal artery	3	Right carotid artery	Yes	41 (36–72)
PTA of superior mesenteric artery	3	Right carotid artery	Yes	23 (22–31)
Left vertebral artery stenting	3	Left femoral artery	Yes	51 (38–58)
Contralateral iliac artery PTA	3	Left femoral artery	Yes	38 (26–53)
Ipsilateral iliac artery stenting	3	Left femoral artery	Yes	18 (14–35)

*Including robotic pre- and post-procedural angiography

PTA, percutaneous transluminal angioplasty

### Procedural Capability and Safety

Technical success was achieved in all 18 interventional procedures (100%), including successful occlusion of the targeted vessels in all coil embolization procedures. No intraoperative complications were observed. Post-procedural angiography demonstrated no signs of vessel injury, including dissection, perforation, distal embolization, or thrombosis. One target vessel spasm was observed in the left renal artery after stenting, which resolved within one hour.

The median procedural times for individual interventions, including angiography, were as follows: left renal artery stenting (38 min; range 38–55 min), embolization of superior branch of right renal artery (41 min; range 36–72 min), PTA of the SMA (23 min; range 22–31 min), left vertebral artery stenting (51 min; range 38–58 min), contralateral iliac artery PTA (38 min; range 26–53 min), and ipsilateral iliac artery stenting (18 min; range 14–35 min).

The estimated median radiation dose that would have been received by an operator positioned at the bedside was 2.03 μSv (range 0.84–4.23) per test subject (with lead protection) and 24.44 μSv (range 13.14–75.44) (without protection) per subject. However, all 18 interventional procedures (100%) were completed entirely via remote operation at the operator-controlled workstation, without the need for manual assistance, resulting in near zero radiation exposure to the operator (0.16 μSv; range 0.14–0.40).

### System Compatibility

The SENTANTE system demonstrated compatibility with a range of standard endovascular devices, including different guidewires (0.014″, 0.018″, and 0.035″), over-the-wire stents, percutaneous transluminal angioplasty balloons, catheters (2.7–8 F), and compatible pushable coils (0.018″ and 0.035″) (Supplemental Tables I and II). Haptic feedback was rated as very good in three test models and as fair in the other three test models.

### Macroscopic Analysis

Macroscopic evaluation focused on assessing the treated vessels and adjacent tissues for signs of procedural trauma. Each vessel was inspected for evidence of tears, ruptures, perforations, dissections, thrombosis, or other structural abnormalities.

Minor vessel trauma was observed in one subject from Group B, consisting of small-caliber-related damage to the left vertebral artery identified during dissection. This finding was attributed to the vessel’s anatomical characteristics rather than any mechanical effect of the robotic system.

In another subject, a focal, annular, segmental red discoloration approximately 1 cm in diameter was observed on the right iliac artery prior to opening. This finding was interpreted as mild intimal damage, a commonly observed finding after catheter manipulation in a porcine model. In the same subject, minimal intimal damage measuring about 1 mm was identified in the left iliac artery during dissection, located 4–5 mm distal to the caudal end of the implanted stent. This was interpreted to be a common, non-flow-limiting dissection following balloon-expandable stent implantation.

## Discussion

This preclinical study demonstrated the feasibility of using the SENTANTE Endovascular Robotic System in performing a variety of endovascular procedures entirely via teleoperation. Across 18 interventions, including stenting, embolization, and percutaneous transluminal angioplasty, technical success was achieved in all cases, with no intraoperative complications observed.

To date, most larger published cohorts on robotic endovascular interventions have focused on PCI, showing comparable results to manual PCI [10, 11]. The Magellan system, which utilizes dedicated wires and steerable catheters, and the CorPath GRX system, compatible with certain off-the-shelf devices, have both demonstrated high procedural success rates during peripheral arterial interventions in case reports, series, and small patient cohorts [6, 12–14]. The Magellan system is equipped with a dedicated self-developed controllable bending catheter, which could be useful in navigating tortuous vessel anatomy and reducing catheter manipulations, potentially lowering the risk of embolization [15, 16]. The downside of requiring dedicated devices is the disruption of the established workflow and increased costs. While the CorPath GRX system was compatible with standard devices, its compatibility was limited, necessitating intermittent manual intervention during the deployment of devices.^7^ Despite their safety and feasibility, both Magellan and CorPath GRX were ultimately discontinued. The SENTANTE system demonstrated compatibility with a broad range of standard vascular devices and successfully completed all procedures in an entirely robotic fashion, other than loading devices on the robot itself.

Vascular interventionalists are among the medical specialists with the highest cumulative occupational radiation exposure [17–19]. While radiation doses typically remain within safety limits, chronic low-dose exposure to ionizing radiation has been associated with an increased risk of malignant neoplasms and vascular disease [20–24]. In the current study, all procedures were completed without the need for intra-procedural manual assistance other than loading devices on the robot itself; therefore, the radiation exposure to the operators was near zero. Across procedures performed with fluoroscopy guidance, radiation use is especially high for embolization procedures and stenting procedures. A robotic system capable of performing these procedures fully robotically may therefore be of particular value in reducing operator radiation exposure. Additionally, the use of a remote workstation eliminates the orthopedic strain associated with lead aprons, thereby improving operators’ ergonomics [25].

Haptic feedback plays an important role in navigating the vascular system, enabling the operator to sense resistance and adjust movements accordingly, reducing the risk of applying excessive force that could damage vessels. The lack of haptic feedback in existing commercial robotic systems may be one of the main limiting factors to their broader uptake in clinical practice [4].

Together with the haptic feedback, the SENTANTE system uses standard wires and catheters as the input tools at the remote workstation. This design allows users to interact with the system in a familiar, intuitive manner. Notably, although the surgeons in the current study had no prior experience with the SENTANTE system, they were able to perform all procedures after 30 min of training.

The future potential of robotic systems in endovascular surgery is substantial. In addition to improving procedural precision, reducing occupational radiation exposure, and serving as a training platform, the use of telesurgery can be a way to operate remotely in rural and underserved areas. Enabling teleoperation to remote areas may prove particularly valuable for specialized procedures with an acute indication, such as mechanical thrombectomy for stroke. Moreover, digital processing of operator input allows for removing natural tremor and rescaling movement of guidewire, enhancing precision and stability during procedures. Also, in working toward partial automation of endovascular procedures, a reliable robotic system represents a foundational step to facilitating this process. Future improvements of the robotic system could involve expanding the range of devices suitable for fully robotic telesurgery, such as self-expanding stent-grafts, and improving procedure times by speeding up the process of exchanging devices and inserting the tools in the vessels once loaded onto the robotic system. The potential benefits, however, need to be weighed against the additional costs to acquire and maintain a robotic system. Given that the SENTANTE system is currently pre-market, the associated costs have not yet been established.

Only six porcine subjects were included in the current study, limiting the statistical power and generalizability of the findings. All procedures were performed in healthy animal models within a controlled GLP-compliant setting, which may not fully replicate real-world hospital conditions or the complexity of diseased human vessels. As the present study is a proof of concept in healthy animal models, no comparison was made to standard manual endovascular procedures and no data was collected on fluoroscopy time, air kerma, and dose area product to facilitate this comparison. Therefore, the performance of the robotic system relative to conventional techniques remains unclear. Future studies are warranted to evaluate the robotic system’s applicability in a clinical setting.

## Conclusions

In a preclinical model, the SENTANTE™ endovascular robotic system demonstrated the feasibility of endovascular interventions entirely via teleoperation, including angiography, stenting, coil embolization, and PTA. Its compatibility with standard endovascular devices, haptic feedback, and elimination of radiation exposure for the operator support progression to clinical feasibility trials.

## Supplementary Information

Below is the link to the electronic supplementary material.

Supplementary file1 (DOCX 21 KB)

Supplementary file2 (MOV 171519 KB)

Supplementary file3 (MOV 135375 KB)
