# Supplementary material for: Evaluation of Fully Teleoperated Robotic Endovascular Interventions with Haptic Feedback: The SENTANTE Endovascular Robotic System
Source: Cardiovasc Intervent Radiol. 2026 Feb 25;49(5):1020–7. doi: 10.1007/s00270-026-04375-w (PMC13156146; doi:10.1007/s00270-026-04375-w)
Supplement: Supplementary file 1 — Supplementary file1 (DOCX 21 KB) [file 270_2026_4375_MOESM1_ESM.docx]

Supplemental Table I. Compatibility of the SENTANTE System with endovascular devices per procedure type for the three study animals in Group A.

| **Target vessel: Renal Artery (left)**  **Procedure: Stenting** | | | | |
| --- | --- | --- | --- | --- |
| **Suggested endovascular devices** | **Brand** | **1** | **2** | **3** |
| Radifocus™ Guide Wire 0.035” | Terumo | X^*^ | X | X |
| Hi-Torque Command 18 ST Guide Wire 0.018” | Abbott | X | X | - |
| V-18^TM^ Control Wire^TM^ 0.018in x 300cm | Boston Scientific | - | - | X |
| Pig Expo^TM^ Catheter | Boston Scientific | X | X | - |
| DxTerity^TM^ Diagnostic Catheter 5-FR | Medtronic | - | X | - |
| Simmons Angiographic Catheter | Terumo | - | - | X |
| Launcher guiding catheter 8-FR | Medtronic | X | - | X |
| Guide catheter Mach1^TM^ | Boston Scientific | - | X | - |
| Vascular Balloon-expandable Stent Formula 418® | Cook Medical | X | X | X |
| **Target vessel: Right Cranial Renal artery branch**  **Procedure: Embolization with coil x 3** | | | | |
| **Suggested endovascular devices** | **Brand** | **1** | **2** | **3** |
| Hi-Torque Command 18 ST Guide Wire 0.018” | Abbott | X | - | - |
| V-18^TM^ Control Wire^TM^ 0.018in x 300cm | Boston Scientific | - | - | X |
| Microcatheter Progreat | Terumo | X | X | X |
| Peripheral coil system AZUR^TM^ 18 4mm x 4cm | Terumo | X | X | X |
| Peripheral coil system AZUR^TM^ 18 3mm x 4cm | Terumo | X | - | X |
| Complex Helical Pushable Coil | Boston Scientific | - | X | - |
| **Target vessel: Superior mesenteric artery**  **Procedure: Percutaneous transluminal angioplasty** | | | | |
| **Suggested endovascular devices** | **Brand** | **1** | **2** | **3** |
| Coaxial catheter system Progreat | Terumo | X | - | - |
| PTA Catheter ARMADA 35 7mm x 20mm x 135cm | Abbott | X | - | - |
| Mustang^TM^ Balloon dilatation catheter 135cm x 8mm x 60mm | Boston Scientific | - | X | - |
| Mustang^TM^ Balloon dilatation catheter 135cm x 8mm x 60mm | Boston Scientific | - | - | X |

* “X” marks the successful advancement and retrieval and full system compatibility with the particular device.

Supplemental Table II. Compatibility of the SENTANTE System with endovascular devices per procedure type for the three study animals in Group B.

| **Target vessel: contralateral iliac (right)**  **Procedure: percutaneous transluminal angioplasty** | | | | |
| --- | --- | --- | --- | --- |
| **Endovascular devices** | **Brand** | **1** | **2** | **3** |
| Launcher guiding catheter 7-FR | Medtronic | X* | - | - |
| Radifocus™ Guide Wire 0.035” | Terumo | X | X | - |
| PTA Catheter ARMADA 35 6mm x 20mm x 135cm | Abbott | X | - | - |
| Pig Expo^TM^ Catheter | Boston Scientific | X | X | X |
| Mustang^TM^ Balloon dilatation catheter 135cm x 7mm x 60mm | Boston Scientific | - | X | X |
| Launcher guiding catheter 8-FR | Medtronic | - | X | - |
| PTA Catheter ARMADA 35 5mm x 20mm x 135cm | Abbott | - | - | X |
| **Target vessel : ipsilateral iliac (left)**  **Procedure: Stenting** | | | | |
| **Endovascular devices** | **Brand** | **1** | **2** | **3** |
| Express^TM^ LD vascular stent system 135cm x 6mm x 27mm | Boston Scientific | X | X | - |
| Radifocus™ Guide Wire 0.035” | Terumo | X | - | - |
| Vascular Balloon-expandable Stent Formula 418® | Cook Medical | - | - | X |
| **Target vessel: Left vertebral artery**  **Procedure: Stenting** | | | | |
| **Endovascular devices** | **Brand** | **1** | **2** | **3** |
| Angiographic Catheter Vertebral | Terumo | X | - | - |
| Neurovascular guide catheter FUBUKI 7-FR | ASAHI | X | - | - |
| RADIFOCUS® Glidewire Advantage^TM^ Guide wire | Terumo | X | - | - |
| Vascular Balloon-expandable Stent Formula 418® | Cook Medical | X | X | X |
| DxTerity^TM^ Diagnostic Catheter 5-FR (JR 5.0) | Medtronic | - | X | X |
| V-18^TM^ Control Wire^TM^ 0.018in x 300cm | Boston Scientific | - | X | - |
| Guide catheter Mach1^TM^ | Boston Scientific | - | - | X |
| Radifocus™ Guide Wire 0.035” | Terumo | - | - | X |
| SAVION DLVR^TM^ Guidewire with ICE^TM^ Hydrophilic coating | Boston Scientific | - | - | X |
| Angiographic Catheter Vertebral | Terumo | - | - | X |

* “X” marks the successful advancement and retrieval and full system compatibility with the particular device.
